# Supplementary figures and images for: Plant different, eat different? Insights from participatory agricultural research
Source: PLoS One. 2022 Mar 25;17(3):e0265947. doi: 10.1371/journal.pone.0265947 (PMC8956185; doi:10.1371/journal.pone.0265947)

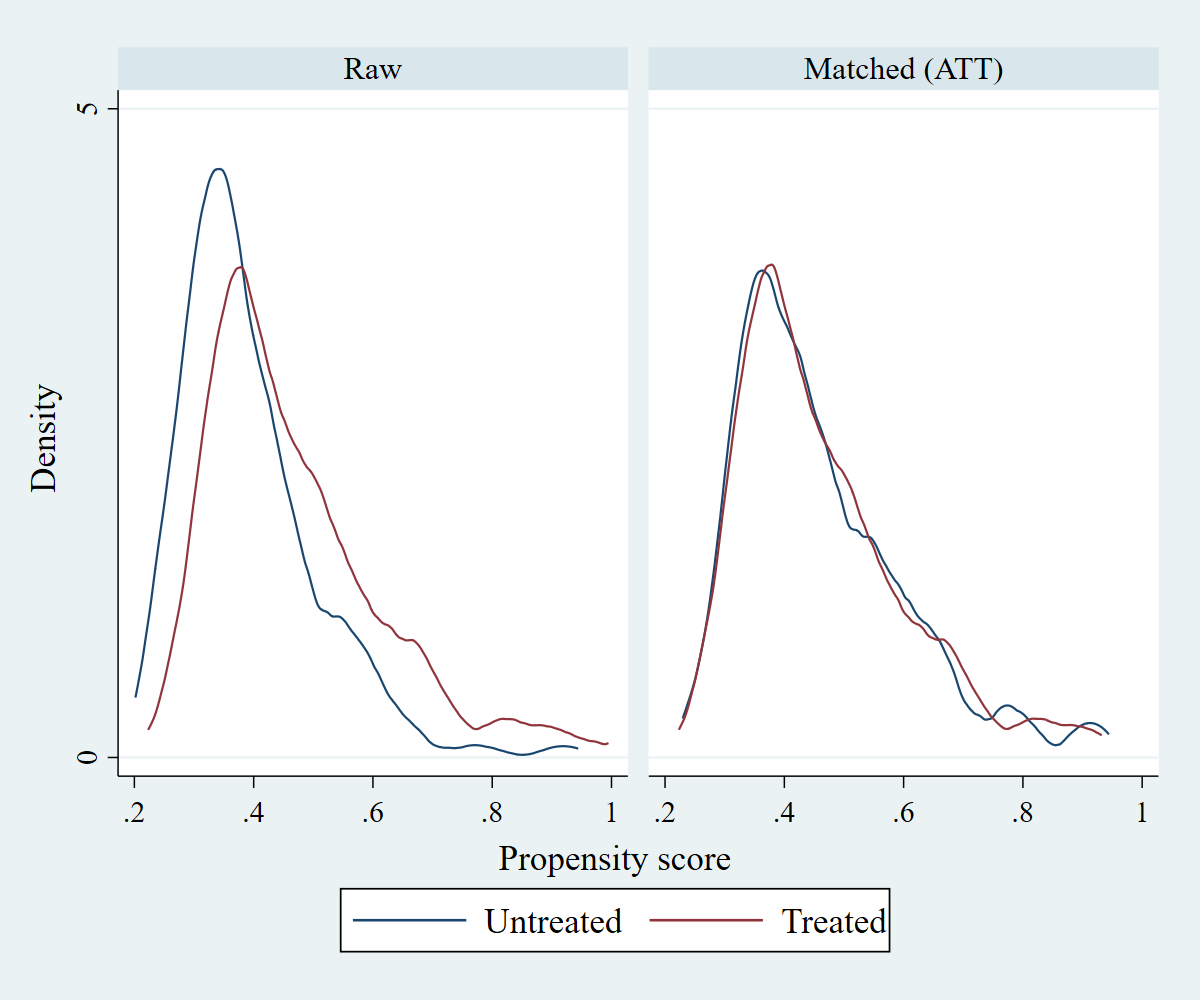

Supplement: S1 Fig — (TIF) [file pone.0265947.s001.tif]

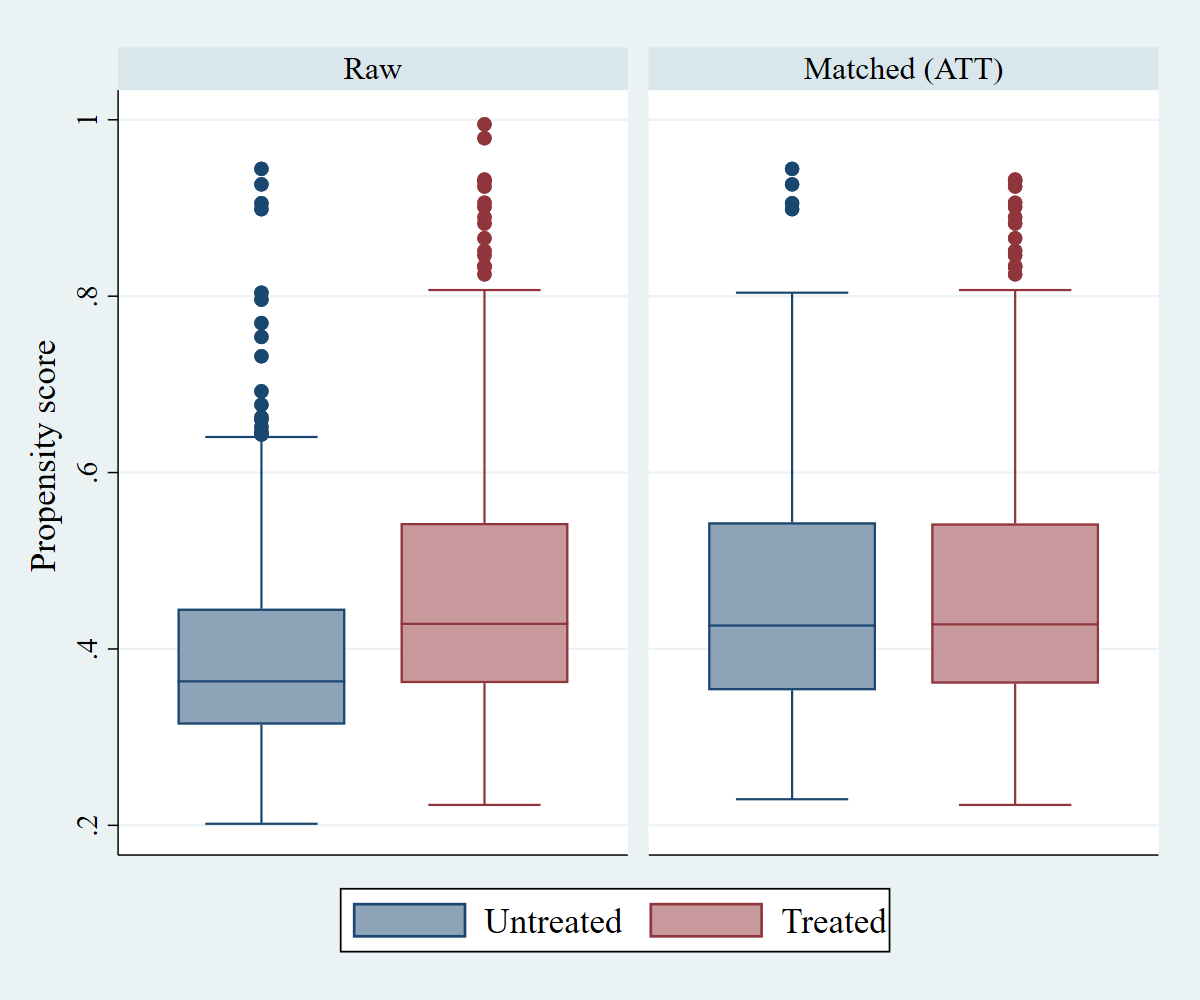

Supplement: S2 Fig — (TIF) [file pone.0265947.s002.tif]
