# Supplementary material for: Plant different, eat different? Insights from participatory agricultural research
Source: PLoS One. 2022 Mar 25;17(3):e0265947. doi: 10.1371/journal.pone.0265947 (PMC8956185; doi:10.1371/journal.pone.0265947)
Supplement: Attachment — Submitted filename: PONE-D-21-30466_Review.docx [file pone.0265947.s009.docx]

**Plant different, eat different? Insights from participatory agricultural research.**

**Reviewer’s Comments**

The manuscript, is an interesting piece of research. This paper analyses the causal effects of on-farm production diversity on household dietary diversity in Malawi based using microdata collected as part of a participatory agricultural intensification program. This is a relevant topic in the body of literature particularly due to the fact that nutrition plays such a significant role human capital development and consequent productivity. Poor nutrition especially for children has long-lasting consequences to the society. After reviewing this paper, I have the following observations concerning the suitability of the article for publishing in the PLOS ONE journal.

In what follows I detail my suggestions for further improvement of the paper.

**Abstract**

Needs minor improvements.

1. The word *commodities* in the sentence “Among other innovations, the program aims to promote the production of pulses, **commodities** that are rich in micronutrients, have better-quality protein, and have nitrogen fixation benefits compared to other grains” can be removed with no alteration in meaning.
2. In the sentence “We find a significant positive **treatment effect** on dietary diversity, led by an increase in production diversity.” The *treatmen*t should be stated directly instead of letting the reader figure it out.

**Introduction**

3. “An important lesson from these studies is that while a diversification strategy has the potential to improve diets and nutrition, the strength of the association is **quantitatively controversial**.” In this statement authors have not stated what these *quantitative controversies* and makes the consequent paragraph on filling in this gap invalid. Authors should clear state what these controversies are and how their study solves those controversies if not justify why and how their study cannot be counted as another adding more controversy.
4. The statement “while **19% and 40%** of the **country’s population and children** below the age of five years are estimated to be **undernourished and stunted**, **respectively**” is not clear whether the figures refer *respectively* to *country population and children* or *undernourished and students.*
5. The sentence “Analysis of the linkage between production diversity and dietary diversity in a crosssectional setting is challenging due to a host of confounding factors …….”

Should be moved from its current paragraph and be used as an opening sentence in the next paragraph

6. The finding that “While production diversification can lead to more diverse diets, increasing production of more nutritious crops such as pulses may not necessarily translate into their greater own consumption, due to the persistence of dietary habits or other location specific factors” should be more elaborately explained; why then should increase in income change dietary diversity despite “the persistence of dietary habits or other location specific factors”

**Materials and methods**

7. Authors need to explain why they have excluded group 2 in the analysis. Obviously if groups 1 and 2 were targeted and only those in group one engaged in the project then you have self-selection. But then the random (as stated) targeting with non-compliance only on the treatment side could provide the Intention To Treat (ITT) which is of interest. In case of non-compliance on both the treatment and the control side the random (as stated) targeting could be used as a clean instrument for participation in the program.
8. Given the nature of the instrument variable (Z) that is used then it makes sense if group 2 is included in the analysis. Because if only groups 1 (program participant) and group 3 (control) are used then there is no way the instrument will be explaining participation (by design) since the control group did not get exposure to the program.
9. Second paragraph of page 6, on-farm production should be removed “Diversity in on-farm production and household diets is measured based on the Simpson’s diversity”
10. There are different measures of production and dietary diversity, authors should argue why they chose to use the Simpson’s index over others.
11. The use of values (instead of quantities) to construct the Simpson’s Index is prone to being affected by food items with high values; authors should also explain why we should not be worried about this. In addition they should also explain how these values were obtained (market price? How about those which were not purchased/sold?)

**Results and Discussion**

12. A number of variables presented in Table 1 could have been affected by the program despite the claim by authors that these variables are unlikely to be affected by the program. For example wealth (measured by assets), dwelling quality, all agricultural inputs and practices variables. Since the program targeted agricultural production, obviously agricultural practices will be affected, incomes and wealth accumulation.
13. Page 14, monitory 🡪 monetary
14. That the claim is the effect ion dietary diversity is mainly through the indirect- channel is through higher agricultural income and food purchasing power. It would be crucial to show empirically whether the program had an effect on household income.

.
