# Supplementary material for: Plant different, eat different? Insights from participatory agricultural research
Source: PLoS One. 2022 Mar 25;17(3):e0265947. doi: 10.1371/journal.pone.0265947 (PMC8956185; doi:10.1371/journal.pone.0265947)
Supplement: Attachment — Submitted filename: Response to Reviewers.docx [file pone.0265947.s010.docx]

*Dear Reviewers:*

*Thank you very much for your detailed comments that provided invaluable feedback on our study. We have revised the manuscript based on your comments and are providing a point-by-point response below (in italics) summarizing revisions to the original manuscript.*

*Best Regards,*

*The authors*

********************************************************************************

# **Reviewer #1: Reviewer’s Comments**

********************************************************************************

The manuscript, is an interesting piece of research. This paper analyses the causal effects of on-farm production diversity on household dietary diversity in Malawi based using microdata collected as part of a participatory agricultural intensification program. This is a relevant topic in the body of literature particularly due to the fact that nutrition plays such a significant role human capital development and consequent productivity. Poor nutrition especially for children has long-lasting consequences to the society. After reviewing this paper, I have the following observations concerning the suitability of the article for publishing in the PLOS ONE journal.
In what follows I detail my suggestions for further improvement of the paper.

Abstract
Needs minor improvements.
1. The word commodities in the sentence “Among other innovations, the program aims to promote the production of pulses, commodities that are rich in micronutrients, have better-quality protein, and have nitrogen fixation benefits compared to other grains” can be removed with no alteration in meaning.

*Response: We have edited the sentence to clarify the focus of the program and to also address your comment #2 below as follows: “The program primarily focuses on the integration of legumes into the cropping system through maize-legume intercropping and legume-legume intercropping. Relative to staple cereals such as maize, legumes are rich in micronutrients, contain better-quality protein, and lead to nitrogen fixation.*

2. In the sentence “We find a significant positive treatment effect on dietary diversity, led by an increase in production diversity.” The treatment should be stated directly instead of letting the reader figure it out.

*Response: Please see response to comment #1 above, in which we clarify the program treatment.*

Introduction
3. “An important lesson from these studies is that while a diversification strategy has the potential to improve diets and nutrition, the strength of the association is quantitatively controversial.” In this statement authors have not stated what these quantitative controversies and makes the consequent paragraph on filling in this gap invalid. Authors should clear state what these controversies are and how their study solves those controversies if not justify why and how their study cannot be counted as another adding more controversy.

*Response: We have removed this sentence and instead cite review studies that summarize key takeaways from previous research including: i) reliance on cross-sectional data to establish associations; ii) mixed evidence on the association between production diversity and dietary diversity not only across but also within countries depending on geography; iii) use of various indicators of production and dietary diversity (Jones 2017; Habtemariam et al. 2021; Sibhatu and Qaim 2018; Sibhatu, Krishna and Qaim 2015). We have also moved the paragraph about IV mediation analysis forward so as to highlight the use of IV mediation analysis in a cross-sectional setting as part of our contribution.*

4. The statement “while 19% and 40% of the country’s population and children below the age of five years are estimated to be undernourished and stunted, respectively” is not clear whether the figures refer respectively to country population and children or undernourished and students.

*Response: We have edited the sentence as follows to avoid confusion: ”…19% of the population is undernourished and 40% of children below five years old are stunted.”*

5. The sentence “Analysis of the linkage between production diversity and dietary diversity in a crosssectional setting is challenging due to a host of confounding factors …….” Should be moved from its current paragraph and be used as an opening sentence in the next paragraph

*Response: We have moved the sentence per your suggestion.*

6. The finding that “While production diversification can lead to more diverse diets, increasing production of more nutritious crops such as pulses may not necessarily translate into their greater own consumption, due to the persistence of dietary habits or other location specific factors” should be more elaborately explained; why then should increase in income change dietary diversity despite “the persistence of dietary habits or other location specific factors”

*Response: We have changed the sentence into: “**while increasing production for markets can lead to more diverse diets* *through increased income that would make affordable an expanded set of food items, increasing production of more nutritious crops such as pulses may not necessarily translate into greater own consumption due to the persistence of dietary habits, tastes, or other local factors that favor consumption of staples such as maize* *and encourage sales of more profitable and nutritious food items such as pulses”. We hope that the revised text clarifies the ideas expressed.*

Materials and methods
7. Authors need to explain why they have excluded group 2 in the analysis. Obviously if groups 1 and 2 were targeted and only those in group one engaged in the project then you have self-selection. But then the random (as stated) targeting with non-compliance only on the treatment side could provide the Intention To Treat (ITT) which is of interest. In case of non-compliance on both the treatment and the control side the random (as stated) targeting could be used as a clean instrument for participation in the program.

*Response: We realized there might be a confusion on this issue in the original manuscript that we have now clarified in the revised version. Group 2 was not targeted and did not benefit from the program at the time of the baseline survey. Households in group 2 just resided in program target villages, and they were included in the baseline survey to measure potential spillovers within program target villages (e.g., through learning effect from households in group 1) at the end of the program by comparing outcomes of groups 2 and those of pure control (group 3). Given the program has expanded its reach within program villages since baseline, it is possible for some households of group 2 to have become program beneficiaries in the meantime. We have updated the paragraph in the data section so that we only talk about the two groups used in our study – beneficiary and control group. We have also cited another study (Haile et al., 2016) that provides additional details about the survey.*

8. Given the nature of the instrument variable (Z) that is used then it makes sense if group 2 is included in the analysis. Because if only groups 1 (program participant) and group 3 (control) are used then there is no way the instrument will be explaining participation (by design) since the control group did not get exposure to the program.

*Response: As mentioned above, including group 2 in the analysis would be justified only in case of partial compliance, but the selection criterion of group 2 was just that households resided in target villages but were not targeted as beneficiaries. We instead use an exogenous instrument (total land area operated by the household that is within 15 minutes of travel from the homestead) as an instrument for program participation, given households with relatively higher access to immediate plots – including experimental ones- were targeted due to need of ease of monitoring by researchers and extension agents. This is in sharp contrast with control households, for whom being exposed to larger cultivated areas close to homestead is a random characteristic.*

9. Second paragraph of page 6, on-farm production should be removed “Diversity in on-farm production and household diets is measured based on the Simpson’s diversity”

*Response: We have edited the sentence to refer to dietary diversity only.*

10. There are different measures of production and dietary diversity, authors should argue why they chose to use the Simpson’s index over others.

*Response: We agree that there are several indices of diversity as summarized in a meta-analysis of the association between production diversity and diets by Sibhatu and Qaim (2018), including simple count of unique food groups as well as a wide variety of indices including Simpson, Shannon, Berger-Parker, Herfindahl, and Margalef. We actually use Simpson and Shannon indices in the analysis -mostly due to their widespread use, simplicity, and ease of interpretation- but decide to keep results based only on Simpson’s in the manuscript since results are qualitatively similar and Simpson’s was used in a previous, cited study on Malawi (Jones, Shrinivas and Bezner-Kerr, 2014).* *One study has noted that while there are several food-group based indicators of dietary diversity, and despite conceptual differences among them, they tend to be positively correlated with each other and with food and nutrition security (Ruel 2003). We have now included results based on Shannon’s diversity index as a sensitivity check (reported in supplemental table S4).* *Simpson’s index is considered more as a dominance index that assigns more weight to dominant groups, while Shannon’s index accounts for both richness and evenness/equitability in distribution and emphasizes the richness component of diversity. In our case, the distribution of both food consumption and agricultural production is skewed towards cereals as we show in supplemental tables S1 and S2.*

*Parameter estimates based on Shannon’s are qualitatively similar to those based on Simpson’s, with expected different magnitude given differences in the values of these indices (**0<Simpson’s<1 and 0<Shannon’s<5). For your information, we are including a table below showing IV mediation results based on the two indices.*

11. The use of values (instead of quantities) to construct the Simpson’s Index is prone to being affected by food items with high values; authors should also explain why we should not be worried about this. In addition they should also explain how these values were obtained (market price? How about those which were not purchased/sold?)

*Response: As you correctly pointed out, Simpson’s production and dietary diversity indices are constructed based on monetary values of total agricultural production and foods consumed inside the household, respectively. Total food consumption expenditure is computed as the sum of self-reported expenditure on purchased food and imputed values of foods consumed from own production or from gifts received. Values of own produced and gifted food are imputed based on quantities and unit values derived by dividing total expenditure on purchased foods by total quantity of purchased foods (see Deaton (1988) for details about unit values). When household surveys include data on food expenditures and physical quantities of foods purchased -as was done in the survey we use-, unit values computed based on purchased foods are used to monetize food consumed from own production. Similarly, we first computed unit values of agricultural commodities produced by dividing total sales revenue by quantities sold and subsequently used commodity-specific unit price values to obtain total value of agricultural production. We have described this strategy in the data section and cited Deaton (1988).*

*We maintain that biases in reported monetary values of food purchases and harvests sold are likely to be less severe than those associated with quantities reported in local units that will first have to be converted into metric units. Given the need to compare production and dietary diversity indices based on the same metrics, we maintain that the decision to use monetary values would likely mitigate eventual measurement error, as outliers would likely be more in numbers and higher in values in distributions expressed in quantities.* *We performed an outlier check and corrected for outliers in monetary values by replacing monetary values higher than +3 Standard Deviations (SD) -or lower than -3 SD- from the median by the distribution median.*

Results and Discussion
12. A number of variables presented in Table 1 could have been affected by the program despite the claim by authors that these variables are unlikely to be affected by the program. For example wealth (measured by assets), dwelling quality, all agricultural inputs and practices variables. Since the program targeted agricultural production, obviously agricultural practices will be affected, incomes and wealth accumulation.

*Response:*

*Thank you for allowing us to clarify this point. Indeed, most of the variables presented in the descriptive statistics Table 1 are not included in the vector of covariates in our IV-mediation analysis that are listed at the bottom of Tables 3 and 4. Our controls include household socio-demographic variables; indices for household dwelling condition; durable agricultural and non-agricultural assets; distance to basic services; self-reported exposure to climatic and crop disease shocks; and finally climatic variables (temperature, rainfall). We control for indices of dwelling condition and durable assets since we believe they were unlikely to have been affected by the program given the short time lapse between program initial enrollment and roll-out (mid-2012) and baseline data capture (mid-2013). We believe that, for example, it was unlikely for program beneficiaries to renovate their dwellings, stock up durable assets, or purchase new land at the time of the baseline, something that might happen over a longer-time horizon. As a sensitivity analysis and robustness check, we have re-estimated the regression models excluding indices of dwelling conditions and durable assets and find results that are qualitatively and quantitatively similar to the original reported. For your information, we present a table below where models in column A are specified as before, while models in column B exclude indices of dwelling condition and durable assets.*

*In addition, given that Table 1 is meant to mimic the traditional balancing t-tests of baseline observables in randomized controlled trials (RCTs), we have now moved variables that capture agricultural technology adoption from panel B of Table 1 to Panel A of Table 2 to avoid confusion. Table 2 provides a descriptive summary of variables likely to have been affected by the program.*

13. Page 14, monitory ◊ monetary

*Response: We have fixed the typo.*

14. That the claim is the effect ion dietary diversity is mainly through the indirect- channel is through higher agricultural income and food purchasing power. It would be crucial to show empirically whether the program had an effect on household income.

*Response: Agricultural income is indeed higher for program beneficiaries based on simple mean comparison reported in Table 2 (panel B). We have now employed statistical matching (kernel with bootstrapped standard errors – 100 repetitions) to estimate average treatment effect on the treated (ATT) and average treatment effect (ATE) and find statistically significant ATT/ATE for all the agricultural outcome variables in Table 2 of Panel B including net agricultural income reported below. Using current exchange rate of 797 Malawian Kwacha to US dollar, the ATT is equivalent to an increase in income by 86 dollars. This result, along with the finding that beneficiaries purchased more diverse foods from the market, is the reason behind the claim about the importance of the indirect, income-related channel.*

| *ATE and ATE estimates on net agricultural income (thousands of Malawi Kwacha)* | | |
| --- | --- | --- |
|  | *ATT* | *ATE* |
|  | *coef/se* | *coef/se* |
|  | *71.689**** | *55.385**** |
|  | *(15.695)* | *(14.282)* |
| *Number of observations* | *915* | *915* |
| *note: .01 - ***; .05 - **; .1 - *;* | |  |

************************************************************************************

# **Reviewer #2: Reviewer’s Comments**

************************************************************************************

Reviewer #2: The paper touches a timely and relevant topic. Although the connection of production and consumption diversity has attracted some attention in the rural development literature, findings on the relationship are mixed. The paper therefore provides a valuable contribution to the existing body of literature. Despite its thematic appeal and well-presented structure - in view of this review - the manuscript should undergo some major revisions to be considered for publication. Main concerns include the claim of causality when interpreting regression results throughout the paper, the suitability of the selected instrument in the regression framework, the lack of background information on the Africa RISING program, as well as the absence of a comprehensive review of the literature on the linkages of production and consumption diversity.

Please find more detailed comments below. Major comments are listed first, minor comments are listed further below. (Line numbering of the manuscript would have been appreciated).

Major comments:
1. Instrumental variable is not convincing. Authors use area of agricultural land within 15 minutes of homestead to instrument household participation in the program. Authors do not argue convincingly why they expect agricultural land within 15 minutes of the homestead to only influence production diversity through program participation. Cropping patterns may differ with increasing distance from a plot to the homestead, e.g., households may cultivate more diverse crops on their plots closer to the homestead - irrespective of program participation. The authors do not present data on production diversity in close to homestead plots for beneficiaries and control households. Authors also do not present information on how the program aimed at increasing production diversity among its beneficiary households. More background information would be needed to support the argumentation for the instrument of choice. In its current narrative, the selection of the IV is not convincing (see also further comments on the IV selection below).

*Response: We thank the reviewer for the arguably central comment, who gives us the opportunity to further clarify and substantiate our choice of instrument. Given the theoretical and empirical requirements for an exogenous, valid, uncorrelated with the outcome, and strong instrument we chose the total land area operated by the household that is within 15 minutes of travel from the homestead. Based on our interactions with program implementers and program site visits earlier on, program participation* *was partly dependent on prospective beneficiary households having as much easily accessible cultivated land close to the homestead as possible for ease of monitoring of research activities by researchers and extension agents alike. Hence, while having as much cultivated land as possible close to the homestead (therefore, easily accessible and with high-intensity management from the farmer) is a characteristic strongly associated to program participants, it is random among control households. Importantly, we also carried out further empirical analysis to reinforce the validity of our IV approach and of the exclusion restriction in particular. This new analysis is described and reported in reply to your comment #18 below.*

*It is possible for cropping patterns to differ with increasing distance from a plot to the homestead irrespective of program participation, although production diversity is statistically higher among program participants, so this means program participation is not independent from production diversity, while belonging to the control group is. Moreover, dietary diversity is only correlated to program participation via production diversity, which is indeed one of the main, explicit goals of the project. As such, our instrument is (strongly) correlated with program participation but uncorrelated with the outcome if not through production diversity, the mediator, satisfying validity. Other econometric conditions (exogeneity and strength) are summarized by the statistical tests provided at the bottom of the tables with regression results.*

2. Claims of causality. The authors employ IV mediation analysis, which is an appealing tool, especially in the context of analysis of complex interactions - here participation in a program, agricultural production diversity, and household dietary diversity. Through its mediation framework, the tool allows the analysis of different pathways from the treatment to the outcome. Authors also provide different mediators and outcomes to complement their analysis. However, in several parts of the paper, authors claim causality. In context of the complex relationship and the previous comment on IV selection, authors should consider to be more cautious in interpretation of results (e.g., by speaking of associations/correlations instead of causation).

*Response: This is indeed a valuable advice. While we believe the identification approach adopted allows us to properly address the identification issues related to selection bias and endogeneity, the reviewer is right in that more caution is warranted in the interpretation of results. We thus followed the advice and removed references to causal relationships, and now mainly talk about significant associations or correlations instead of causal claims when interpreting and discussing the results. Below we report some instances of these replacements:*

*p.7: “The goal of mediation analysis is to unpack the transmission mechanisms in which T and M jointly cause Y, by disentangling the total effect of T (TE) on Y into two components: the indirect or mediated effect, that is the effect of T on Y that operates exclusively through its effect on M; and the direct effect, that is the residual ~~causal~~ effect of T on Y that is not mediated by M, that is holding the distribution of M constant.”*

*p.8: “This approach allows the use of the same instrumental variable to identify the ~~causal~~ complex chain among the outcomes within a standard IV strategy that controls for treatment endogeneity with respect to the intermediate and final outcomes.”*

*p.21: “We investigate the ~~causal chain~~ statistical associations between production diversity and dietary diversity in Malawi using cross-sectional household survey data collected as part of a sustainable intensification agriculture research program.”*

*p.22: “The positive ~~causal effect~~ and significant correlations between production diversity ~~on~~ and diversity of purchased food highlights the importance of access to food markets for increasing and reinforcing nutritional gains associated to enhanced on-farm production diversity.”*

*Finally, we also added the following paragraph in the conclusions:*

*“**Lastly, some caveats are necessary regarding the interpretation of our findings. While the novel empirical approach adopted allows us to overcome econometric challenges related to selection bias, endogeneity, simultaneity, and the cross-sectional nature of the data, the results provided here should nonetheless be interpreted with caution: the significant associations and correlations found are suggestive of causal relationships, but causal claims will have to be more thoroughly supported by new empirical evidence when follow-up data finally become available.”*

3. Framing of the story. The authors frame the story around PP – PD – DD (program participation- production diversity-dietary diversity). Yet, authors results (and interpretation thereof) indicate that positive associations between PP and DD are not necessarily associated with PD per se, but with increases in maize output and income, which in turn is used to increase HH DD. Authors claim in some parts of the manuscript that PD leads to higher DD, which should be rephrased to avoid misinterpretation.

*Response: We think the main, overarching narrative is around PP-DD, with the mediation analysis used to disentangle how much of the DD increase is due directly to PD or indirectly through income increase. In this respect, the framing of the story around PP-PD-DD is only part of the narrative, the other part being PP-income increase-DD.*

*Agricultural income is indeed higher for program beneficiaries based on simple mean comparison reported in Table 2 (panel B). We have now employed statistical matching (kernel with bootstrapped standard errors – 100 repetitions) to estimate average treatment effect on the treated (ATT) and average treatment effect (ATE) and find statistically significant ATT/ATE for all the agricultural outcome variables in Table 2 of Panel B including net agricultural income reported below. Using current exchange rate of 797 Malawian Kwacha to US dollar, the ATT is equivalent to an increase in income by 86 dollars.*

| *ATE and ATE estimates on net agricultural income (thousands of Malawi Kwacha)* | | |
| --- | --- | --- |
|  | *ATT* | *ATE* |
|  | *coef/se* | *coef/se* |
|  | *71.689**** | *55.385**** |
|  | *(15.695)* | *(14.282)* |
| *Number of observations* | *915* | *915* |
| *note: .01 - ***; .05 - **; .1 - *;* | |  |

*This result, along with the finding that beneficiaries purchased more diverse foods from the market, is the reason behind the claim about the importance of the indirect, income-related channel that we hope to have clarified in the revised manuscript.*

4. Lack of background information on Africa RISING. Although the program is introduced briefly in the paper, the authors may want to provide more information on Africa RISING. In its current form, reader does not learn what the program does in terms of promotion of legume and/or maize production, when interventions started, from when the data presented stems, how beneficiaries were selected (targeted?) into the program, intervention logic, and modes of implementation. The presentation of program objectives, mode of beneficiary selection, location, timing, and concrete interventions are essential to contextualize the findings of regression results.

*Response: We have added a section on study setting before the materials and methods section also based on the reviewer’s comment #9 below. The subsection provides a high-level summary of Malawian agriculture and details about the sustainable intensification program (timeline, targeting, interventions, and expected outcomes) we relied on in the empirical analysis.*

5. Lack of comprehensive review of literature examining linkages between production and consumption diversity. Although authors briefly refer to existing literature on the topic (e.g., last sentence of last paragraph of introduction p.3 and last sentence of following paragraph, p.3), the manuscript does not provide a comprehensive review of their findings. Authors should include such a review – also highlighting that findings are inconclusive and inconsistent, also pointing to the fact that the relationship (production diversity and consumption diversity) is likely to be highly complex and context dependent.

*Response: We have now revised the sentences and cited review studies that summarize key takeaways from previous research including: i) reliance on cross-sectional data to establish associations; ii) mixed evidence on the association between production diversity and dietary diversity not only across but also within countries depending on geography; iv) use of various indicators of production and dietary diversity* (Jones 2017; Habtemariam et al. 2021; Sibhatu and Qaim 2018; Sibhatu, Krishna and Qaim 2015)*. We have also moved the paragraph about IV medication analysis forward so as to highlight the use of IV mediation analysis in a cross-sectional setting as part of our contribution.*

6. P.4 “We tackled these challenges by using household survey microdata […]”. Authors correctly point to the challenges associated with the examination of linkages between production and consumption diversity in the previous sentence. The cited sentence claims to present how these problems are tackled, yet it only presents the data source.

*Response: Thank you for spotting the inconsistency: the two sentences were indeed unrelated. The whole paragraph has been re-arranged with the discussion about challenges in establishing linkages moved to the introduction section while details about the data are provided in the data and key variables section.*

7. P.5 “In this study we use recently developed mediation analysis […]”. Authors may want to include a reference in relation to the method, as well as references in which the method has been applied in similar contexts

*Response: We have added the following sentence in the introduction section: “…**we use the recently developed mediation analysis approach applied to instrumental variables (IV) frameworks (Dippel, Ferrara and Heblich 2020; Dippel, Gold, et al. 2020) in which multiple endogeneity and transmission mechanisms are simultaneously at play and are controlled for using a single instrument. The approach has been successfully employed in several recent publications (Dippel, Greif and Trefler 2020; Dippel et al. 2021; Lin et al. 2021).”*

8. P.5 “The underlying mechanism, however, appears to be through higher purchase of more diverse food […]” and “[…] these effects are primarily due to an increase in the production of a secondary target crop, maize, and not to higher pulse production.” Considering this finding, authors may want to rephrase statements in the manuscript which may be misinterpreted by readers in the sense that diversification of production leads to diversification in production. E.g., abstract: “These findings highlight that, while diversifying production can lead to more diverse diets […]”.

*Response: We agree with the reviewer and have changed the sentence in the abstract into “while increasing production for markets can lead to more diverse diets through increased income that would make affordable an expanded set of food items,…”. We have also revised the conclusions.*

9. P.5 Before Materials and Methods section, authors may consider providing background information on Africa RISING (also see comment #4).

*Response: We have now included a section on study setting as noted in our response to reviewer’s comment #4 above.*

10. P.6 “This study analyzes data […]”. Please explain why group 2 households were not considered in the analysis.

*Response: Group 2 was not targeted and did not benefit from the program at the time of the baseline survey. Households in group 2 just resided in program target villages, and they were included in the baseline survey only to measure potential spillovers within program target villages (e.g., through learning effect from households in group 1) at the end of the program by comparing outcomes of groups 2 and those of pure control (group 3). Including group 2 in the analysis would be justified only in case of program partial compliance, but the selection criterion of group 2 was just that households should reside in target villages but were not targeted as beneficiaries. We have updated the paragraph so that we only talk about the two groups used in this study to avoid confusion. We have cited another paper (Haile et al. 2016) where readers can find additional details about the survey design.*

11. P.6 “Agricultural production data refer to […]”. Please briefly discuss advantages and disadvantage of 7-day recall approach. Please also reflect on how the harvest cycle/period of cultivated crops coincides with the concrete dates of the recall period. That is can households consume own produce in the 7-day period prior to interview.

*Response: Maize harvesting period is between mid-April and beginning of October (between June and July for pigeon pea) for the long rainy season in medium-altitude areas in Malawi (where most of our sample is located), according to the FAO cropping calendar. Given our survey has been conducted between August and October 2013, food consumption data recall period largely coincides with the harvesting time. Therefore, sampled households can consume own-produced food during the 7-day period prior to the interview.*

12. P.7 Identification. Please define T, M and Y as used in the manuscript, when mentioned for the first time.

*Response: Thank you. We have revised the paragraph as follows:*

*“The goal of mediation analysis is to unpack the transmission mechanisms in which a treatment T and a mediator M jointly cause an outcome of interest Y, by disentangling the total effect of T on Y into two components: the indirect or mediated effect, that is the effect of T on Y that operates exclusively through its effect on M; and the direct effect, that is the residual effect of T on Y that is not mediated by M, that is holding the distribution of M constant.”*

13. P.8 “[…] validity, exogeneity, and strength [of the IV]”, replace by relevance, exclusion restriction, and independence assumption?

*Response: Words have been replaced, thanks.*

14. P.9 “We posit the partial identifying assumption to hold in our setting, […]”. This assumption merits further discussion. E.g., what variables do authors expect to jointly affect T and PD (to confound DD through PD)? Why do authors expect other confounders of DD (not through PD) to be of less importance?

*Response: Thank you for this comment, which gives us the opportunity to better clarify why we deem this novel empirical approach is suitable in this complex setting. We have re-arranged and extended the paragraph as follows:*

*“**In our case, this approach allows us to investigate the production-consumption transmission chain while accounting for non-random selection into the program, endogeneity, and potential simultaneity between production and consumption decisions. In this complex transmission chain, we assess the effect of participation in the Africa RISING program (T) on the Simpson’s household dietary diversity index (Y) as mediated by the effect of T on another mediator outcome (M), the Simpson’s on-farm production diversity index. We posit the partial identifying assumption to hold in our setting, as it is plausible to assume that program participation is endogenous in a regression of dietary diversity (DD) on T only due to confounders that jointly affect T and production diversity (PD). As the explicit goal of the program was to diversify agricultural production, there are only two main channels through which the treatment (participation) could affect the dietary diversity of beneficiary households: either by increasing their production diversity through the cultivation of new cash crops, thus raising their agricultural income and, in turn, their consumption of more diverse foods; or by diversifying the variety of subsistence crops and, in turn, directly boost their dietary diversity. Both these channels are mediated by agricultural production diversity. For these reasons, which are strictly related to the nature and design of the project, we can safely assume that T is endogenous with respect to DD due to confounders that affect DD primarily through PD.”*

15. P.9-10 “[…] we estimate Equations 5 and 6 using 2SLS […]”. Considering the complex relationship between PD and DD, and the IV at hand, estimation of the causal chain seems quite ambitious. Authors may consider referring to associations instead of causal chains.

*Response: We agree with the reviewer and have revised the paragraph as follows:*

*“Adapting the formalization of the IV mediation analysis framework to our study, we estimate Equations 5 and 6 using 2SLS to examine the associations between program participation, production diversity, and dietary diversity.”*

16. P.10 “Our choice of an exogenous instrument for program participation is guided by program design, […]”. As noted in an earlier comment, the manuscript does not contain much information on program design. Please include, also to support your argument for the IV.

*Response: We chose the total land area operated by the household that is within 15 minutes of travel from the homestead since one of the informal criteria for program participation was having as much easily accessible cultivated land close to the homestead as possible. This criterion was mostly due to ease of agricultural monitoring by researchers and extension agents alike. Hence, while having as much cultivated land as possible close to the homestead (therefore, easily accessible and with high-intensity management from the farmer) is a characteristic strongly associated to program participants, it is totally random among control households. We have now added more details on the program design under study. Please also refer to our response to your comment #18 below for results from the additional analysis we have conducted to validate our choice of instrument.*

17. P.10 “Specifically, we use total land area operated by the household […]”. It would be interesting to see whether there are differences in cropping patterns on these plots between participants and non-participants.

*Response: Please see our response to your comment #18 below where we have conducted a multivariate analysis that indirectly validates the exclusion restriction assumption. We have added a paragraph in the identification section describing the placebo test we have conducted that provides indirect empirical evidence in favor of the exclusion restriction with test results reported in supplemental table S5.*

18. P.10 “[…] land area within 15 minutes travel would not affect DD unless through program participation.” This is a strong statement and goes along with major comment #1. There may be channels other than program participation through which land area within 15 minutes of homestead may affect DD. E.g., if households tend to cultivate certain crops more often on plots closer to their home, and if these crops tend to be marketed/ consumed more often, DD could also be affected by (pre-existing) cropping patterns on these plots, irrespective of program participation.

*Response: We thank the reviewer for this comment, with is related to comment #1 above. We have removed this sentence and instead included a paragraph about the placebo test we have conducted to provide indirect empirical evidence in support of the exclusion restriction. Specifically, in the revised version we now present a placebo test -in which we compare the reduced form regressions for the intermediate outcome (production diversity)- run first on the whole sample and then only on the untreated sample. The test shows that while the instrument is positively and significantly associated with the intermediate outcome in the main reduced form, the coefficient turns close to zero and statistically insignificant in the reduced form regression in the untreated subsample. Please find the new supplemental table S5 below -also reported in the Appendix- as well as the new text in the revised version that discusses the placebo test results (p.18).*

**Table S5**

| Placebo test – Reduced form regressions for the intermediate outcome | | |
| --- | --- | --- |
|  | (1) | (2) |
|  | Simpson’s production diversity | |
|  | Full sample | Control group |
| Area of household plots within 15 minutes of travel | 0.0104** | 0.0013 |
|  | -0.00413 | -0.00737 |
| Observations | 935 | 538 |
| Control variables | Yes | Yes |
| Note: Standard errors clustered at the household level are reported in parentheses. *** p<0.01, ** p<0.05, * p<0.1. | | |

*“**We provide indirect empirical evidence in favor of the exclusion restriction of our chosen instrument (area of household plots within 15 minutes of travel). Specifically, we carry out a placebo test in which we run two reduced form regressions of our IV approach for the intermediate outcome (production diversity), one on the whole sample and another one on the untreated group only. As the latter is by definition not affected by the treatment, we expect to find a significant association between production diversity and the instrument in the first case (for the whole sample) and a null effect in the second case (for the untreated group)”. Indeed, Table S5 in the Appendix shows this result.*

*“While the instrument is significantly associated with the intermediate outcome in the main reduced form, the coefficient turns substantially smaller and statistically insignificant in the reduced form regression of the untreated subsample. This discrepancy suggests that the instrument has no overall effect on production diversity for the subsample of households that did not participate in the sample, and no direct effect on it other than through the treatment itself. As our approach assumes that the treatment is endogenous with respect to the final outcome (dietary diversity) due to confounders that also jointly affect agricultural production, and since the smaller substitution effect detected in the mediation analysis also stems from changes in crop production patterns, this test provides strong indirect support for the validity of the exclusion restriction”.*

19. Page 11 The reference to Fig. 1 appears twice.

*Response: We have fixed the references.*

20. P.12 “Since survey data were collected […]. Please indicate when data was collected and when program activities started.

*Response: We have changed the sentence into: “Household survey data were collected between August and October 2013 after program beneficiaries obtained their first harvest since joining the program”*

21. P. 13 Table 1. Number of observations (n=935) does not match with the total number of observations presented in the data section (N=1149). Please explain.

*Response: We realized there might be a confusion on this issue in the original manuscript that we have now clarified in the revised version. The reason why the number of observations in the regression (n=935) and the total number of observations presented in the data section (n=1149) differ is because we excluded households belonging to group 2 in the multivariate analysis. This exclusion was due to the fact that household in group 2 were not targeted and did not benefit from the program at the time of the baseline survey. Households in group 2 just resided in program target villages, and they were included in the baseline survey to measure potential spillovers within program target villages (e.g., through learning effect from households in group 1) at the end of the program by comparing outcomes of groups 2 and those of pure control (group 3). As such, the multivariate analysis is based on household survey data from 397 program beneficiary and 538 control households. We have updated the paragraph in the data section so that we only talk about the two groups used in our study – beneficiary and control group. We have also cited another study (Haile et al., 2016) that provides additional details about the survey.*

22. P. 14 Table 2. Table 2 shows that beneficiaries tend to have higher maize yields, higher value of maize harvested, and higher net agricultural income with relative importance of income from maize >> income from legumes. How does maize production fit into the assessed relationship between production diversity and dietary diversity? From the manuscript, it seems like the program mainly tries to introduce additional legumes into household production. What is the role of maize production?

*Response: We have added more details about the program in the newly added Study Setting subsection. In addition to crop-livestock integration and integration of pulses, the program has encouraged the use of fertilizers, plant spacing, and row planting, testing different fertilization rates, spacing measures, and plant densities to increase maize productivity and, hence, production. In addition to their nutritional benefits, pulses utilize soil bacteria to draw nitrogen from the air (known as nitrogen fixation) thereby replacing the need add nitrogen fertilizers in pulse fields and crops intercropped wit pulses (e.g., maize)*

23. P.15 “As noted in previous studies, […]”. The authors may want to contextualize their findings regarding the sources included in the cited literature (e.g., sources 1-7). E.g., literature analyzing linkages between on -farm production diversity and household consumption diversity has produced mixed findings: Ecker (2018) finds that production diversity is associated with HH consumption diversity mainly through direct pathways (HH consuming own produce), while (Sibhatu et al. 2015)find that increase in HH consumption diversity is associated with increased HH income (and not by consumption of own produce).

*Response: Thank you. We have cited these studies in the discussion section.*

24. P.16 “This result suggests that the market channel is more important […]”. Please also put into context of literature on PD and DD.

*Response: We have added “in line with previous findings -Sibhatu et al (2015)-“ to better contextualize the result.*

25. P.22 “Results point to an overall positive impact of the program on dietary diversity […]”. In the context of results presented earlier (direct effect being outsized by indirect effect), this formulation may be misleading. Consider rephrasing.

*Response: Agree with the reviewer. The sentence was unclear and, for this reason, it did not convey what we really meant, i.e., that we do find beneficial effects of participation to the program on dietary diversity, and that this result is mainly driven by the indirect effect. Following the reviewer’s advice, the paragraph has been revised as follows:*

*“Results point to a positive and significant impact of the program on dietary diversity, which is mainly driven by the increase in production diversity of beneficiary households. In sum, participation led to a more diverse agricultural production and, through this mediating channel, to an increase in dietary diversity.”*

26. P.22 “Enhanced maize production boosts both own-consumption and, perhaps more importantly, agricultural income, allowing households to purchase and consume more diverse food items.” Would this finding not imply that PD does not play a large role in DD. After all, Maize is the most grown cash crop in Malawi, and as such not associated with PD (or is it?). How would T (program participation)->M (production diversity)->Y (dietary diversification) hold in this case? What does the program promote in terms of maize production? Does PD even matter?

*Response: Yes, we largely agree with the reviewer. Results points to an overall positive impact of the program on dietary diversity of beneficiary households, mostly driven by the propulsive role of income increase that we also document based on statistical matching we implemented (see comment #3 above). However, given the key mandate and activities of the program towards judicious use of fertilizer and improved seeds in maize cultivation, as well as maize-legume and short-term legume with long-term legume intercropping to increase not only production diversity but also boost maize production, we found that the indirect role of legumes in increasing maize production through nitrogen fixation is crucial. That is, our findings point to the indirect role of production diversity (the mediator) on dietary diversity through income increase due to legumes and maize sales. Under this mechanism, production diversity does mostly matter indirectly.*

27. Supplementary Material. Results from models S1(1b) and Table 3 (1) are not the same, which they should (same T, M, and Y). Please explain, also why number of observations is not the same for both models (S1 n=931, table 1 n=935).

*Response: We believe you meant S3(1b) not S1(1b). As noted in Equations 1 – 4 of our paper, as well as in Equations 7-10 of the methodological paper on IV mediation analysis we rely on (Dippel, Ferrara, et al. 2020), the IV mediation analysis can be formalized within the standard 2SLS framework and involves intermediate results reported in Table S3(1b), along with the main results presented in Table 3 (1). The intermediate results reported includes the following results I (where Y is outcome, T is treatment, Z is instrument, and M is mediator):*

*R1. IV regression of Y on T (where T is instrumented with Z). Please note that we have not reported R1.*

*R2. IV regression of M on T (where T is instrumented with Z), for which the ﬁrst-stage F-statistic is reported as stage one in the main table. This is what we reported in S3(1a).*

*R3. IV regression of Y on M (where M is instrumented with Z) controlling for T, for which the ﬁrst-stage F- statistic is reported as stage two in the main table. This is what we reported in S3(1b)*, *when dietary diversity is measured based on all foods, as well as when dietary diversity is measured based on purchased foods only -in S3(1c)-.*

*The association between the main results (total, direct, indirect) reported in Table 3 (1) and intermediate results presented in S3(1b) for completeness is as follows:*

- *The total effect is given by the coefficient of T in R1*
- *The direct effect is given by the coefficient of T in R3*
- *The indirect effect is given by the product of the coefficient of T in R2 and the coefficient of M in R3.*

*For example, focusing on dietary diversity based on all foods consumed (Table S3, column 1b):*

- *The coefficient of T (= -0.094) is the same as the direct effect reported in the main table – Table 3 (1).*
- *The product of the coefficient of T reported in Table S3 1a (0.415) and the coefficient of M in S3 1b (0.926) equals 0.38, which is the same as the indirect effect presented in Table 3 (1).*

*We have updated the note below Table S3 to emphasize the link between the supplemental results and main results along the lines illustrated above.*

************************************************************************************

# **Reviewer #3: Reviewer’s Comments**

************************************************************************************

The study design (cross-sectional survey) does not seem to adequately measure program participation, and it seems unlikely that there would be significant impacts on production after just 1 year of the study, so the overall results are not very compelling or convincing. The authors need to provide more information about the intervention and study design to be able to justify their conclusions. Further, there are several recent studies in Malawi that are not included in the paper and the authors reference to this as an ‘agricultural intensification’ study seems at odds with the diversification emphasis. Finally, gender dynamics in agriculture need to be adequately addressed in the paper.

Introduction:

- The authors conclude from their brief review of the linkages between diversified farming systems and nutritional outcomes is that ‘An important lesson from these studies is that while a diversification strategy has the potential to improve diets and nutrition, the strength of the association is quantitatively controversial.’ [italics added]. My understanding of the literature is somewhat different from this conclusion. Recent reviews of a considerable literature on this subject have all shown a positive, significant association between production diversity and dietary diversity for farming households, including 2 reviews included in the literature review (Jones 2017; Sibhatu et al. 2015). The question is perhaps to what extent other factors matter more or mediate that relationship, and several studies have pointed to the significance of factors such as gender relations in mediating that relationship, including studies done in Malawi (e.g. Bezner Kerr et al. 2019).

*Response: The papers cited by the reviewer offer a mix of findings. Jones (2017) concludes that “agricultural biodiversity has a small but consistent association with more diverse household- and individual-level diets, although the magnitude of this association varies with the extent of existing diversification of farms.” Taking the case of four countries (Indonesia, Malawi, Kenya, and Ethiopia) Sibhatu et al. (2015) highlight that “the link between farm production diversity and dietary diversity does not universally exist…”. They also add that “increasing on-farm diversity is not always the most effective way to improve dietary diversity in smallholder households and should not be considered a goal in itself.*” *Hence, even from just these two studies, the relationship is either non-existent or, if it exists, is associated to a small effect. We have cited additional review studies that summarize key takeaways from previous research pointing towards mixed results including: i) reliance on cross-sectional data to establish associations; ii) mixed evidence on the association between production diversity and dietary diversity not only across but also within countries depending on geography; iii) use of various indicators of production and dietary diversity (Jones 2017; Habtemariam et al. 2021; Sibhatu and Qaim 2018; Sibhatu, Krishna and Qaim 2015). We have also moved the paragraph about IV mediation analysis forward so as to highlight the use of IV mediation analysis in a cross-sectional setting as part of our contribution.*

*Yes, as the reviewer also suggests, our analysis aims at exploring to what extent other factors matter more or mediate the relationship (provided that it exists and is strong, which is also something our study is set to examine), and this is the reason why we use mediation analysis in the multivariate framework that takes account of endogeneity. Unfortunately, given the data at hand we cannot study gender-mediated relations as done by Bezner-Kerr et al. 2019 -for the case of participatory agroecological research on climate change adaptation-, which is something we would like to explore in future research.*

- The authors refer to their intervention as ‘agricultural intensification’ but from the description the intervention appears to be crop diversification. Crop/farm diversification is often contrasted to intensification in the literature, because intensification usually entails monocropping and increased input use rather than diversification. Diversification, in contrast, or diversified farming systems, is often linked to an agroecological approach and has been a subject of policy debate for farming and food systems because of many environmental services as well as food security and nutritional outcomes. This broader debate within which diversification of food systems sits might be briefly noted in the literature review. Some possible references:

*Response: We agree with the reviewer in that intensification is often linked to monocropping and increased input use rather than crop/farm diversification. What we mean throughout our manuscript is sustainable agricultural intensification (SAI), which indeed entails crop/farm diversification, crop-livestock integration, minimal use of fertilizers, careful residue management, minimal soil disturbance, optimal plant spacing and density, various intercropping methods between cereals and legumes, as well as among different types of legumes. We have now added the sustainability dimension throughout the revised manuscript, as the program under analysis is indeed a participatory sustainable intervention, similar in objectives and implementation to an agroecological approach.*

o Bezner Kerr, R. et al. 2021. Can agroecology improve food security and nutrition? A review. Global Food Security 29. <https://doi.org/10.1016/j.gfs.2021.100540>
o Rasmussen, L.V., et al. 2018. Social-ecological outcomes of agricultural intensification. Nat. Sustain. 1, 275–282. <https://doi.org/10.1038/s41893-018-0070-8>

- The authors could integrate more recent studies that examine these relationships in Malawi or similar contexts that were not included in this review:

*Response: We have added the recent references on agroecology and sustainable intensification kindly suggested by the reviewer in the introduction and study setting sections as appropriate*

Madsen, S. et al. 2021. Explaining the impact of agroecology on farm-level transitions to food security in Malawi. Food Security 13: 933–954.

Kansanga, M.M. et al. 2021. Agroecology and household production diversity and dietary diversity: Evidence from a five-year agroecological intervention in rural Malawi. Social Science and Medicine 288, 113550. <https://doi.org/10.1016/J.SOCSCIMED.2020.113550>

Santoso, M.V. et al. 2021. A nutrition-sensitive agroecology intervention in rural Tanzania increases children’s dietary diversity and household food security but does not change child anthropometry: results from a cluster-randomized trial. Journal of Nutrition.
doi:https://doi.org/10.1093/jn/nxab052

Bezner Kerr, R. et al. 2019. Participatory agroecological research on climate change adaptation improves smallholder farmer household food security and dietary diversity in Malawi. Agriculture, Ecosystems and Environment 279: 109-121. <https://doi.org/10.1016/j.agee.2019.04.004>

Snapp, S. S.,&Fisher, M. (2015“. "filling the maize bas”et" supports crop diversity and quality of household diet in Malawi. Food Security, 7(1), 83–96.

Methods:

There is very limited information about the intervention. Who was involved, and what support was provided to the household? How was it participatory?

*Response: We have now included more details about the program under the new “study setting” section added. The reference about “participatory” specifically refers to a research design known as ‘mother-and-baby’ trials involving the creation of interactive and replicable demonstration plots known as mother-trial plots centered around farmer action groups. Group members subsequently set up adaptive trials known as baby-trials to test a subset of technologies demonstrated on the mother trials.*

How was participation measured in the study – simply that they were enrolled? In which case how was lack of participation taken into account? Other mediation studies have much more detailed information about the intervention in order to determine the overall relationships, eg. Cetrone et al. 2021. Food security mediates the decrease in wo’en's depressive symptoms in a participatory nutrition-sensitive agroecology intervention in rural Tanzania. Public Health Nutr. 24(14):4682-4692. doi:10.1017/S1368980021001014. Epub 2021 Mar 12. PMID: 33706829.

*Response: We hope that the additional details on the specific interventions and its main delivery methods provided in the revised manuscript clarify how participation was measured. To add to that, participation was identified listing all the farmers who were directly interacting with the implementers on the ground and were exposed to the interventions through demonstration, field days, or “model” farmers. Unfortunately, we do not have data on non-compliers nor information on technology disadopters that might have changed our econometric strategy.*

Results

Given that this study took place only 1 growing season after the intervention began, it seems somewhat premature to anticipate impacts on dietary diversity from production diversity – it may take 2 or more growing seasons for farmers to realize some of the longer term impacts from diversified production systems, including production as well as improved soil fertility, reduced pests and diseases etc. In addition they may not be able to produce significant enough yield of the new crop in the first season to generate enough for sale, seed and consumption and so may prioritize one of those options. These aspects need to be discussed in relation to your results.

*Response: Yes, we agree with the reviewer that it might be premature to assess impact of production diversity on dietary diversity just one agricultural season after the start of the intervention. Indeed, our estimates need to be viewed as initial forecast to be validated with follow-up data once they are collected and become available. Therefore, our companion future paper will validate the results presented here with the use of additional data at the end of the intervention to take all the cited elements (improved soil fertility, reduced pests and diseases, enhanced market sales and consumption, even eventual shift in diets given the longer term elapsed) into account. Indeed, one important caveat in the conclusions is that “…the effects we document may be limited to the short-run due to limited time elapsed between beginning of the program and data collection -just one completed cropping season-.[…] Therefore, a key area for future research is a longitudinal analysis of longer-term effects of the program to examine whether and to what extent gains in knowledge, adoption of improved innovations, and environmental services can bring about longer-term effects on production and consumption patterns of beneficiary households.”*

The authors state that ‘This result suggests that the market channel is more important than the increase in food own-consumed generated by enhanced production diversity..’. This is a cross-sectional study, so the survey results do not demonstrate that there has been ‘enhanced production diversity’ only that participants grow more food, they might have been doing so prior to the intervention.

*Response: Yes, we agree with the reviewer that this is a cross-sectional study, so we cannot explore the variation of specific variables over time. However, our study is set to explore the static relationship between production and dietary diversity at one point in time, taking advantage of the spatial, household-level variation rather than the additional time variation as in a panel setting. In this respect, results of our mediation IV analysis should be interpreted as LATE (local average treatment effect) model estimates stemming from application of non-experimental methods rather than experimental methods, such as difference-in-difference on panel RCT data. We have now rephrased the sentence into: “This result suggests that the market channel is more important than higher food own-consumed generated by greater production diversity among beneficiary households (in line with previous findings -Sibhatu et al (2015)-“, to better contextualize this finding.*

The authors also seem to somewhat misrepresent the findings of a review on agricultural diversification and dietary diversity. They indicate that “on-farm diversity has a small and nonlinear association with dietary diversity” and cite Jones (2017). The nonlinear association is true, pointing to several different pathways discussed in the review by Jones (2017), but note that the author found that: “agricultural biodiversity has a small but consistent association with more diverse household- and individual-level diets, although the magnitude of this association varies with the extent of existing diversification of farms. Greater on-farm crop species richness is also associated with small, positive increments in young child linear stature.”

*Response: We have removed this sentence and added key takeaways from existing literature (see comment above on the Introduction) including: i) reliance on cross-sectional data to establish associations; ii) mixed evidence on the association between production diversity and dietary diversity not only across but also within countries depending on geography; iii) use of various indicators of production and dietary diversity (Jones 2017; Habtemariam et al. 2021; Sibhatu and Qaim 2018; Sibhatu, Krishna and Qaim 2015).*

Discussion

The authors seem to mention gender issues as an afterthought at the end of the section, and only in relation to involving both men and women. Previous research in Malawi and in other places has demonstrated that unequal gender dynamics within households can also influence dietary outcomes, such that increased production diversity may not translate into consumption of the food products (see Ruel and Alderman 2013 for example). Addressing gender dynamics as part of a nutrition-sensitive intervention has also been shown to increase the likelihood of improved dietary diversity (e.g. Bezner Kerr et al. 2019; Santoso et al. 2021). this aspect of gender inequality is not discussed in the paper and does not seem to have been taken into account adequately in the study design or analytical methods.

*Response: We agree with the reviewer that, unfortunately, given the data at hand we cannot study gender-mediated relations as done by other literature. We feel that unpacking gender dynamics in assessing the relationship between production and dietary diversity due to an agricultural technology participatory program would be the topic of a whole new paper. Our study examines the relationship between the two variables due to agriculture-related, income, and consumption dimensions, regardless of different social constructs and other confounding factors intervening in the relationship. Nevertheless, as mentioned, in our future companion paper we will look at gender-related dynamics taking advantage of the panel follow-up survey data when available.*

Ruel MT, Alderman H. Nutrition-sensitive interventions and programmes: How can they help to accelerate progress in improving maternal and child nutrition? Lancet 2013;382(9891):536–51.

The authors state that “Our results point towards the importance of increasing the productivity and profitability ‒ including of Malawi’s main staple crop maize ‒ to enhance market purchasing power.” Please explain how your results point to the importance of increasing maize production – given the dominance of maize in production and consumption, it is not clear how your results point to such a finding.

*Response: The paper shows that program participation has a positive effect on maize yield, value of maize and pulses produced, and agricultural income. Participants also report higher crop sales, both in levels and as share of total harvest, showing higher output market integration and purchases of more diverse foods from the market as shown in Tables 3 and 4 in the manuscript. Even agricultural income is indeed higher for program beneficiaries based on simple mean comparison reported in Table 2 (panel B). We have now employed statistical matching (kernel with bootstrapped standard errors – 100 repetitions) to estimate average treatment effect on the treated (ATT) and average treatment effect (ATE) and find statistically significant ATT/ATE for all the agricultural outcome variables in Table 2 of Panel B including net agricultural income reported below. Using current exchange rate of 797 Malawian Kwacha to US dollar, the ATT is equivalent to an increase in income by 86 dollars. This result, along with the finding that beneficiaries purchased more diverse foods from the market, is the reason behind the claim about the importance of the indirect, income-related channel.*

| *ATE and ATE estimates on net agricultural income (thousands of Malawi Kwacha)* | | |
| --- | --- | --- |
|  | *ATT* | *ATE* |
|  | *coef/se* | *coef/se* |
|  | *71.689**** | *55.385**** |
|  | *(15.695)* | *(14.282)* |
| *Number of observations* | *915* | *915* |
| *note: .01 - ***; .05 - **; .1 - *;* | |  |

*We have edited the paragraph by specifying these results.*

They also state that “Efforts aiming at reducing barriers to better market integration (e.g., limited information about prices and high transportation costs) could enhance participation in profitable markets and boost household dietary diversity.” The results presented do not appear to say anything about market integration, so it is not clear how these conclusions are drawn. Could not better market integration increase consumption of unhealthy imported purchased foods, as observed in many other parts of the Global South?

*Response: Yes, we agree with the reviewer that higher market integration might increase consumption of unhealthy purchased food. However, the reference to market integration in this case is specific to integration into output markets. We have clarified this feature in the manuscript. Simply put, given that effects on dietary diversity stem mostly from household consumption of diverse food items purchased from the market, improved market access would be beneficial to the household given the enhanced market opportunities and, hence, greater market sales. Then, to assess the global, net effects of higher market integration, a different (computable partial, or even general equilibrium) model should be used instead of (or in addition to) our econometric analysis.*

The authors go on to state: “On the other hand, these market-oriented actions should be complemented with an active soil fertility monitoring, aimed at increasing nutritional content of crops that are otherwise sourced from the market with a consequent welfare-decreasing effect. Hence, the initial specialization strategy in the main staple crop should be accompanied by a diversification strategy to the extent that the new legume crops attain desirable physical properties to be able to substitute market-sources commodities.”
The results presented do not appear to provide any information about soil fertility or nutritional content of crops, so again it is not clear how such conclusions are drawn.

*Response: Yes, it is true that results presented are not directly linked to soil fertility and crop content. However, in the sentence cited we try to provide some policy suggestions and actions given the overall results found. The reference to soil fertility and nutritional content of crops is mostly drawn from authors’ knowledge about possible supportive measures that would be consistent with the findings, given the local context, mostly characterized by maize specialization. By contrast, we are suggesting to also explore a specialization strategy, according to a similar recent study by (Bellon et al. 2020) on the same program and topic.*

It is not clear how an intensification strategy would be accompanied by a diversification strategy since these often work at cross purposes, as a number of studies of the FISP program demonstrates:

*Response: As mentioned earlier, intensification needs to be complemented by the sustainability dimension, as SAI is perfectly consistent with a diversification strategy. Indeed, SAI lies on crop/farm diversification, crop-livestock integration, and on various intercropping methods between cereals and legumes, as well as among different types of legumes. As previously highlighted, we have now added the environmental sustainability dimension throughout the revised manuscript.*

Chibwana, C., Fisher, M., Shively, G., 2012. Cropland Allocation Effects of Agricultural Input Subsidies in Malawi. World Dev. 40, 124–133. <https://doi.org/10.1016/j.worlddev.2011.04.022>

The authors note that this is surprising, but based on my own knowledge and understanding of FISP from long-term research in Malawi, while legumes are officially a part of the program, they constitute a much smaller proportion of the total amount of seeds distributed, and the emphasis is on maize production.

*Response: Yes, we agree on the official versus the actual efforts and initiatives of the FISP, more towards maize production in the end (as witnessed by our sentence “While agricultural interventions in Malawi have historically targeted maize”).*

# References

Bellon, M.R., B.H. Kotu, C. Azzarri, and F. Caracciolo. 2020. “To diversify or not to diversify, that is the question. Pursuing agricultural development for smallholder farmers in marginal areas of Ghana.” *World Development* 125:104682. Available at: https://doi.org/10.1016/j.worlddev.2019.104682.

Deaton, A. 1988. “Quality, Quantity, and Spatial Variation of Price.” *American Economic Review* 78(3):418–430. Available at: http://www.jstor.org/stable/1809142%5Cnhttp://www.jstor.org/stable/pdfplus/1809142.pdf?acceptTC=true.

Dippel, C., A. Ferrara, and S. Heblich. 2020. “Causal mediation analysis in instrumental-variables regressions.” *Stata Journal* 20(3):613–626.

Dippel, C., R. Gold, S. Heblich, and R. Pinto. 2020. “Mediation Analysis in IV Settings With a Single Instrument.” *Unpublished manuscript*.

Dippel, C., R. Gold, S. Heblich, and R. Pinto. 2021. “The Effect of Trade on Workers and Voters.” *The Economic Journal.* 132(641):199–217.

Dippel, C., A. Greif, and D. Trefler. 2020. “Outside options, coercion and wages: Removing the sugar coating.” *The Economic Journal* 130(630):1678–1714.

Habtemariam, L.T., C. Gornott, H. Hoffmann, and S. Sieber. 2021. “Farm Production Diversity and Household Dietary Diversity: Panel Data Evidence From Rural Households in Tanzania.” *Frontiers in Sustainable Food Systems* 5(May):1–10.

Haile, B., C. Azzarri, C. Roberts, and D.J. Speilman. 2016. “Targeting, Bias, and Expected Impact of Complex Innovations on Developing-country Agriculture: Evidence from Malawi.” *Agricultural Economics*.

Jones, A.D. 2017. “Critical review of the emerging research evidence on agricultural biodiversity, diet diversity, and nutritional status in low- and middle-income countries.” *Nutrition Reviews* 75(10):769–782.

Jones, A.D., A. Shrinivas, and R. Bezner-Kerr. 2014. “Farm production diversity is associated with greater household dietary diversity in Malawi: Findings from nationally representative data.” *Food Policy* 46:1–12.

Lin, C., C. Ma, Y. Sun, and Y. Xu. 2021. “The telegraph and modern banking development, 1881–1936.” *Journal of Financial Economics* 141(2):730–749.

Ruel, M. 2003. “Operationalizing Dietary Diversity: A Review of Measurement Issues and Research Priorities.” *J Nutr.* 133(11 Suppl 2):3927S–3931S.

Sibhatu, K.T., V. V. Krishna, and M. Qaim. 2015. “Production diversity and dietary diversity in smallholder farm households.” *Proceedings of the National Academy of Sciences of the United States of America* 112(34):10657–10662.

Sibhatu, K.T., and M. Qaim. 2018. “Review: The association between production diversity, diets, and nutrition in smallholder farm households.” *Food Policy* 77(October 2017):1–18. Available at: https://doi.org/10.1016/j.foodpol.2018.04.013.
