# Supplementary material for: Plant different, eat different? Insights from participatory agricultural research
Source: PLoS One. 2022 Mar 25;17(3):e0265947. doi: 10.1371/journal.pone.0265947 (PMC8956185; doi:10.1371/journal.pone.0265947)
Supplement: Attachment — Submitted filename: Response to Reviewers-Feb192022.docx [file pone.0265947.s011.docx]

*Dear Reviewer,*

*Thank you very much for your feedback on the revised paper. We have made further revisions to address your pending comments. Below, we provide point-by-point response (in italics) summarizing revisions to the manuscript.*

*Best Regards,*

*The authors*

Reviewer #2: Thanks to the authors for their detailed responses to the comments. Most of the remarks made during the first review have been fully addressed. In particular, the manuscript now contains a section on program details, authors have altered wordings in relation to claims of causal relation and have linked their findings on the relation of production and dietary diversity to relevant literature. The manuscript continues to be well-structured, well-written and comprehensive in terms of data presentation and analysis.

Despite the reviews undertaken, the central comment during the first review – the choice of the instrumental variable – has only been addressed partially. Please find the main arguments against the IV in its current way of presentation below. Being fully aware of the complexity of discussions about IV selection, some suggestions to support the current IV are provided for the authors consideration.

1. Choice of IV.
It is acknowledged that authors have put great effort into the selection of an appropriate instrument for their analysis. The new manuscript contains additional tests (placebo test) in the supplementary material, and a comprehensive discussion of its results and implications for the analysis in the main body of the manuscript. Authors have also revised the Identification chapter and provided a theoretical background on IV choice in IV mediation analysis, based on which authors have motivated their IV choice. The arguments presented by the authors can be followed, and the instrument is certainly a suitable predictor for program participation (T) – as it was a precondition to be selected as beneficiary.

Yet the presented discussion and results are not (yet) conclusive regarding the overall suitability of the IV. This concerns mainly the exclusion restriction. Authors use land within 15 minutes of homestead to instrument program participation. In the context of smallholder agriculture, land holdings and locations of land holdings may be correlated with several (unobserved) factors that may simultaneously also influence crop and livestock production patterns, management practices, yield levels, output marketing, all of which ultimately may influence HH consumption decisions. In its current form, the manuscript does not show convincingly that treatment and control households do not differ systematically with respect to some unobserved features that influence both size of land holdings within 15 minutes of the homestead (the precondition to receive treatment) and the outcome (dietary diversity). This is not trivial, as descriptive comparison of treatment and control groups presented in tables 1 and 2, shows significant differences across several variables, e.g., treatment households tend to be better educated, less female led, have larger total land holdings, are less likely to be poor, and own more livestock. While it can be argued that certain differences are associated with the treatment itself, especially Simpson’s production diversity index and marketed value of legumes, other differences such as households’ land holdings, location of landholdings, and poverty incidence (based on both durable non-agricultural assets, and on dwelling condition) are likely independent of the treatment and therefore pre-existing. Thus, it cannot be ruled out that households with relatively more land holdings within 15 minutes of the homestead (essentially the treatment households) systematically share unobserved characteristics that affect their wealth status, consumption expenditures and dietary diversity. In this case, estimates of treatment effect on DD would be biased, most likely upward biased.

*Response:* *Thank you for your detailed notes related to the instrument used in the IV mediation analysis – total land area within 15 minutes of travel from the homestead (hereafter, nearby). We would like to put forward some considerations to your attention. Having a nearby land was not a condition that was explicitly used to target program beneficiaries. That is, it was not a* “precondition to receive treatment”. *Please also note that variables reported in Table 2 are indeed indicators expected to differ by treatment status, which include use of intercropping, harvest value of program-target crops, production diversity, and household food consumption.*

*We have conducted additional data analyses, reporting two summary tables below to better motivate our choice of instrument. We have updated the identification section of the manuscript accordingly. Summary Table 1 shows that the share of nearby agricultural parcels owned by households is lower than that of faraway parcels for both treated and control groups. This finding may be due to shortage of agricultural land near residential areas (affecting whether a household owns a nearby plot or not – effect at the extensive margin). The difference in the share of nearby vs faraway parcels is higher for beneficiaries (48% nearby vs 53% faraway) than for the control group (38% nearby vs 63% faraway).*

*Table 1. Summary of parcel distribution by treatment status and parcel location*

| *Group* | *# of parcels* | *parcels <=15 minutes of travel* | |  |  | *parcels >15 minutes of travel* | |
| --- | --- | --- | --- | --- | --- | --- | --- |
|  |  | *#* | *%* |  |  | *#* | *%* |
| *Beneficiary* | *1079* | *517* | *48* |  |  | *562* | *52* |
| *Control* | *1008* | *378* | *38* |  |  | *630* | *63* |

*Summary Table 2 shows that nearby parcels are, on average, smaller than faraway parcels. Once again, this could be due to shortage of agricultural land near residential areas, which affects the size of parcels conditional on owning one– effect at the intensive margin. We do not find statistically significant differences in several agronomic indicators by parcel location for the whole sample, but instead observe differences in selected indicators by parcel location and treatment status. Specifically, beneficiaries grow higher # of unique crops per parcel and are more likely to practice cereal-legume intercropping on faraway plots relative to nearby plots, while the opposite holds for control households. This result may be due to the fact that beneficiaries needed to devote at least 10 square meters to each program legume-related technology (or a mix thereof)* (Haile et al. 2016)*. This constraint, coupled with the fact that faraway parcels were, on average, bigger than nearby parcels may have resulted in beneficiaries testing program target technologies -including intercropping- on faraway parcels.*

*Table 2. Selected agronomic practices and outcomes by parcel location and treatment status*

Authors have responded, with inter alia, a placebo test. As stated by the authors, this test provides indicative evidence that the size of land holdings within 15 minutes of the homestead seems to affect production diversity only through program participation (treatment) as the reduced form model yields an insignificant coefficient for the regression of PD on landholdings within 15 minutes of the homestead among control households. Thus, land holdings as such are not likely to directly affect production diversity of a given household. Yet, the results do not show whether there are any links between landholdings within 15 minutes of the homestead and dietary diversity other than through production diversity. In other words, while the test provides strong evidence for the instrument’s relevance, it does not provide support for the exclusion restriction to hold (the main critique of this comment).

To provide additional support for their choice of IV, authors may consider running the placebo test directly on the outcome (not on the mediator) to test whether the exclusion restriction holds (IV only affects DD through PD). For the treatment group the coefficient of such a test would indicate the “[..] effect of T on Y that operates exclusively through its effect on M” (citation from manuscript p.14). For the control group, the coefficient should not be statistically different from zero, as the only way through which landholdings close to the homestead would affect DD should be through PD (T). Results could support the statement made on p. 16 “we can safely assume that T is endogenous with respect to DD
due to confounders that affect DD primarily through PD”.

*Response: We have conducted additional placebo tests running three reduced form regressions of the final outcome (household dietary diversity) separately for overall, treated, and control samples. Results are shown in Table S5 (also reported below), along with reduced form results on the intermediate outcome (production diversity). The coefficient of the instrument is positive and significant for beneficiaries (and the whole sample), but not for the control group suggesting, as you correctly expected, that the instrument does not affect dietary diversity for control households that have not benefited from program interventions.*

S5 Table. Placebo tests – Reduced form regressions on the intermediate and final outcomes

|  | (1) | (2) | (3) |  | (4) | (5) | (6) |
| --- | --- | --- | --- | --- | --- | --- | --- |
|  | Simpson's production  diversity index | | |  | Simpson's dietary  diversity index | | |
|  | Full sample | Beneficiary | Control |  | Full sample | Beneficiary | Control |
| Total area of nearby plots | 0.007** | 0.008** | -0.003 |  | 0.011** | 0.008** | 0.012 |
|  | (0.003) | (0.003) | (0.008) |  | (0.004) | (0.004) | (0.008) |
| Control variables | Yes | Yes | Yes |  | Yes | Yes | Yes |
| Observations | 935 | 397 | 538 |  | 935 | 397 | 538 |
| Note: Reduced form estimates from linear regression of Simpson's production diversity (columns 1-3) and household dietary diversity (columns 4-6) indices are reported, controlling for the instrument and other covariates that include household size, age and gender of the household head, average education of adults, dependency ratio, land size, indices for durable agricultural and non-agricultural assets, dwelling conditions, access to basic services, temperature, rainfall, and fixed effects for Extension Planning Area (EPA). Cluster-robust standard errors in parentheses. *** p<0.01, ** p<0.05, * p<0.1. | | | | | | | |

In addition, authors may consider running a (supplementary) propensity score matching, using available control variables, especially land holdings, wealth status, as matching parameters to estimate the ATE and ATT of the treatment on dietary diversity. Although bias due to unobserved pre-treatment differences may still not be ruled out, PSM would ensure that treatment effects are assessed based on similar HH in control and treatment groups, and magnitude of results could be compared with those of IV mediation analysis. In this context, authors mays consider using sensitivity analysis to test for unobserved confounders.

*Response: We have estimated treatment effect on the treated (ATT) and average treatment effect (ATE) using inverse probability weighting with regression adjustment (IPWRA)* (Cattaneo 2010)*. IPWRA allows to address the endogeneity associated with self-selection into treatment by modelling both treatment selection and outcome variables, which is particularly relevant in the context of non-random treatment assignments as is the case in this study. Our correction related to observable differences are based on Equations 1 and 2 that we have estimated using probit (for treatment, Equation 1) and ordinary least squares (for household dietary diversity, Equation 2).*

$Pr(T_{h}=1) =g\left( \boldsymbol{Z}_{h}; \boldsymbol{\theta} \right)+ \varphi_{h}$	*(1)*

$y_{h}=f\left( \boldsymbol{X}_{h}; \boldsymbol{\beta} \right)+ \varepsilon_{h}$	*(2)*

*where* $T_{h}=1$ *if* $h$ *is a beneficiary and*$T_{h}=0$ *otherwise;* $g\left( \cdot\right)$ *and* $f\left( \cdot\right)$ *are assumed functional forms;* $\boldsymbol{Z}$ *now includes household demographic characteristics, land holdings, indices for durable agricultural and non-agricultural assets, wealth status (variables you have suggested to include), as well as climatic variables; and* ***X*** *is a vector of covariates affecting* $y$ *(some of which overlapping with those included in* ***Z****)*$.$ *In our case,* $\boldsymbol{X}$ *includes variables in* ***Z*** *as well as Simpson’s production diversity to minimize omitted variables bias in the dietary diversity model in Equation 2 and to mimic the IV mediation analysis where the intermediate outcome is production diversity. Parameters* $\boldsymbol{\beta}$ *and* $\boldsymbol{\theta}$ *are vectors of unknown parameters to be estimated;* $\varphi$ *is selection model error term assumed to have a normal distribution;* $\varepsilon$ *is outcome model error assumed to be independent and identically distributed (i.i.d.) with zero conditional mean. IPWRA estimator is said to be “doubly robust”, meaning that only one of the two models (treatment or outcome) must be correctly specified to consistently estimate treatment effects* (Bang and Robins 2005)*. IPWRA is consistent if either* $f\left( \cdot\right)$ *or* $g\left( \cdot\right)$ *is correct and is more efficient, especially relative to weighting adjustment, if* $f\left( \cdot\right)$ *is correct* (Cattaneo 2010; Zhao 2004)*. While the conditional mean independence assumption for matching is inherently untestable, we assess the quality of the match by examining the balance of covariate distribution by treatment status. Results are reported in supplemental table S7, also reported below for your reference. While only ATT is marginally significant (at 10% level) when looking at Simpson’s dietary diversity based on all foods consumed (column 1), both ATT and ATE are significant when Simpson’s dietary diversity is measured using purchased foods only (columns 3 and 4), although ATE is only marginally significant (column 4). ATT stronger effect on diversity of purchased foods in consistent with results from the IV mediation analysis reported in Table 3 where approximately two-thirds of the increase in the Simpson’s dietary diversity index -computed based on all foods consumed inside the household- was due to increase in the diversity of food purchased from the market.*

S6 Table. ATE and ATE estimates on Simpson’s production and dietary diversity indices

|  | Simpson's dietary diversity: all foods | |  | Simpson's dietary diversity: purchased foods | |
| --- | --- | --- | --- | --- | --- |
|  | (1) | (2) |  | (3) | (4) |
|  | ATT | ATE |  | ATT | ATE |
|  | coef/se | coef/se |  | coef/se | coef/se |
|  | 0.04* | 0.01 |  | 0.08*** | 0.04** |
|  | (0.03) | (0.02) |  | (0.03) | (0.02) |
| Observations | 926 | 926 |  | 926 | 926 |
| Note: Average treatment effect on the treated (ATT) and average treated effect (ATE) on Simpson's household dietary diversity index are reported, based on inverse probability weighted regression adjustment estimator (IPWRA). Columns 1 and 2 show results based on all foods consumed inside the household while columns 3 and 4 show results based on purchased foods consumed. Bootstrapped standard errors (with 100 repetitions) in parentheses.  *** p<0.01, ** p<0.05, * p<0.1. | | | | | |

*As we discussed in the paper, the magnitude of impact estimates based on IPWRA is nonetheless smaller than that from IV mediation analysis. While both IV and risk adjustment (RA) approaches such as IPWRA are designed to mitigate confounding bias using non-experimental methods to estimate causal impact, the two approaches are not directly comparable and IV impact estimates cannot be truly interpreted as ATT or ATE* (Fang, Brooks and Chrischilles 2011)*. When treatment effects are homogeneous across the target population or, if heterogeneous, are unrelated to treatment assignment, RA and IV estimates produce comparable results when their associated identifying assumptions hold. On the other hand, when treatment effects are heterogeneous and are related to treatment assignment, the two approaches may produce asymptotically different estimates. Treatment effect estimates from RA and IV often differ in the same study due to differences in the measurement of associations in IV-based approaches and in the type of treatment effects estimated in RA-based approaches* (Heckman and Robb 1985; Brooks and Chrischilles 2007; Fang et al. 2011)*.*

*To assess the overall quality of the match, we considered the Rubin’s B and R statistics, along with the thresholds outlined in* *Rubin (2001).* *Rubin's R refers to the ratio of beneficiary to control variances of the propensity scores, while Rubin’s B refers to the absolute standardized difference in the means of the propensity score between beneficiary and control groups. Rubin’s R statistic should be below 2 to avoid over-correction of bias and above 0.5 to prevent under-correction, while Rubin’s B should be below 25. As shown below, Rubin’s B and R after matching are within the recommended thresholds.* *Ultimately, the estimation sample for the treated versus control group comprises a sub-sample of matched households that fall within the common support (921 households of which 394 are beneficiaries).*

Matching analysis statistics for bias (B) and the ratio of variances (R):

|  | Rubin's B | Rubin's R |
| --- | --- | --- |
| Unmatched | 56.60 | 1.83 |
| Matched | 11.89 | 1.08 |
| Rubin statistics thresholds: B<25% - R inside [0.5; 2] | | |

*S1 Fig. Box plots of propensity scores before and after matching*

*S2 Fig. Kernel density plots of propensity scores before and after matching*

Additional minor comments
2. Introduction and study setting.
Authors may consider sharpening the introduction and study setting chapters, by focusing on the relationship of diversification of agricultural production and household nutrition. Especially the opening sentences of the study setting chapter feel out of context (although content is certainly relevant for the study). Authors may consider opening the study setting chapter with an overview of the status of farms’ production diversity (or lack thereof) and continue with a description of how the objectives of the program under study aim at enhancing production diversity and what are expected outcomes (e.g., diversifying maize production).

*Response: We have added the following discussion to the introduction section to highlight the importance of market access in mediating the interaction between production and dietary diversity:*

*Especially when access to markets to buy food and sell agricultural commodities is limited, diverse agricultural production can play a vital role in ensuring diversified diets* (Koppmair, Kassie and Qaim 2017)*. For example, evidence from Ethiopia shows that households that live far away from market centers not only consumed less diverse foods but also had lower food consumption expenditure relative to households who live close to markets* (Usman and Callo-Concha 2021)*.”*

*At the beginning of the introductory paragraph -under the study setting subsection- we have added the following sentence: “Malawi’s crop production is highly dominated by continuous maize production. For example, a recent study based on a panel of field-level data from Central and Southern Malawi shows that maize was continuously grown for four years on more than 55% of the plots in the Central region and more than 82% of the plots in the Southern region* (Burke, Jayne and Snapp 2022)*. This practice has implications not only for depletion of essential soil nutrients but also for diversity of household diets.”*

*To improve flow, we have also moved this sentence: “[…] maize is Malawi’s main staple food accounting for about half of the total plant-based caloric intake with pulses and groundnut accounting for just 7%* (FAO 2013)*….” to the end of the paragraph after noting that the intended focus of the government’s input subsidy program was initially on maize, although it was later expanded to legumes, recognizing the latter’s contribution to improving soil quality and nutrition.*

3. Page 6 first paragraph.
The preceding paragraph states that findings are mixed across and within studies. It is not evident how the cited examples for South America support this contradiction of findings (tuber are promoted and consumed more often; livestock ownership leads to greater animal sourced food consumption).

*Response: We have removed the following sentence since, as you correctly pointed out, it is not proof of the fact that the evidence if mixed. “For example, participants of an agricultural program in South America that promoted native grains and tubers increased consumption of the same* (Bellon, Gotor and Caracciolo 2015)*, while livestock ownership (e.g., cows, poultry, and small ruminants) was associated with higher ASF consumption and better child nutrition* (Hoddinott et al. 2015; Azzarri et al. 2015; Bhagowalia, Headey and Kadiyala 2012)*.” Instead, we have cited review papers* (Jones 2017; Habtemariam et al. 2021; Sibhatu and Qaim 2018; Sibhatu, Krishna and Qaim 2015) *where the reader can find more information about the mixed evidence on the association between production and dietary diversity.*

*References*

Azzarri, C., A. Zezza, B. Haile, and E. Cross. 2015. “Does Livestock Ownership Affect Animal Source Foods Consumption and Child Nutritional Status? Evidence from Rural Uganda.” *Journal of Development Studies* 51(8):1034–1059.

Bang, H., and J.M. Robins. 2005. “Doubly robust estimation in missing data and causal inference models.” *Biometrics* 61(4):962–973.

Bellon, M.R., E. Gotor, and F. Caracciolo. 2015. “Assessing the effectiveness of projects supporting on-farm conservation of native crops: Evidence from the high andes of South America.” *World Development* 70:162–176.

Bhagowalia, P., D.D. Headey, and S. Kadiyala. 2012. “Agriculture, income, and nutrition linkages in India: Insights from a nationally representative survey.” *IFPRI Discussion Paper 01195*.

Brooks, J.M., and E.A. Chrischilles. 2007. “Heterogeneity and the interpretation of treatment effect estimates from risk adjustment and instrumental variable methods.” *Medical Care* 45(10):130.

Burke, W.J., T.S. Jayne, and S.S. Snapp. 2022. “Nitrogen efficiency by soil quality and management regimes on Malawi farms: Can fertilizer use remain profitable?” *World Development* 152:105792. Available at: https://doi.org/10.1016/j.worlddev.2021.105792.

Cattaneo, M.D. 2010. “Efficient semiparametric estimation of multi-valued treatment effects under ignorability $.” *Journal of Econometrics* 155:138–154.

Fang, G., J.M. Brooks, and E.A. Chrischilles. 2011. “Apples and oranges? Interpretations of risk adjustment and instrumental variable estimates of intended treatment effects using observational data.” *American Journal of Epidemiology* 175(1):60–65.

FAO. 2013. “Malawi: BEFS Country Brief.”

Habtemariam, L.T., C. Gornott, H. Hoffmann, and S. Sieber. 2021. “Farm production diversity and household dietary diversity: Panel data evidence from rural households in Tanzania.” *Frontiers in Sustainable Food Systems* 5(May):1–10.

Haile, B., C. Azzarri, C. Roberts, and D.J. Speilman. 2016. “Targeting, bias, and expected impact of complex innovations on developing-country agriculture: Evidence from Malawi.” *Agricultural Economics*.

Heckman, J., and R. Robb. 1985. “Alternative methods for evaluating the impact of interventions: An overview.” *Journal of Econometrics* 30(1–2):239–267.

Hoddinott, J., D. Headey, M. Dereje, J. Hoddinott, D. Headey, and M. Dereje. 2015. “Cows, Missing Milk Markets, and Nutrition in Rural Ethiopia Cows, Missing Milk Markets, and Nutrition in Rural Ethiopia.” *The Journal of Development Studies* 51(8):958–975.

Jones, A.D. 2017. “Critical review of the emerging research evidence on agricultural biodiversity, diet diversity, and nutritional status in low- and middle-income countries.” *Nutrition Reviews* 75(10):769–782.

Koppmair, S., M. Kassie, and M. Qaim. 2017. “Farm production, market access and dietary diversity in Malawi.” *Public Health Nutrition* 20(2):325–335.

Rubin, D. 2001. “Using propensity scores tohelp design observational studies: application to the tobacco litigation.” *Health Services and Outcomes Research Methodology* 2:169–188.

Sibhatu, K.T., V. V. Krishna, and M. Qaim. 2015. “Production diversity and dietary diversity in smallholder farm households.” *Proceedings of the National Academy of Sciences of the United States of America* 112(34):10657–10662.

Sibhatu, K.T., and M. Qaim. 2018. “Review: The association between production diversity, diets, and nutrition in smallholder farm households.” *Food Policy* 77(October 2017):1–18. Available at: https://doi.org/10.1016/j.foodpol.2018.04.013.

Usman, M.A., and D. Callo-Concha. 2021. “Does market access improve dietary diversity and food security? Evidence from Southwestern Ethiopian smallholder coffee producers.” *Agricultural and Food Economics* 9(1).

Zhao, Z. 2004. “Using matching to estimate treatment effects: Data requirements, matching metrics, and Monte Carlo evidence.” In *Review of Economics and Statistics*. MIT Press 238 Main St., Suite 500, Cambridge, MA 02142-1046 USA journals-info@mit.edu , pp. 91–107.
